# Perspectives on the Current State of Bioprinted Skin Substitutes for Wound Healing

**DOI:** 10.3390/biomedicines11102678

**Published:** 2023-09-29

**Authors:** Celena A. Sörgel, Aijia Cai, Rafael Schmid, Raymund E. Horch

**Affiliations:** Laboratory for Tissue Engineering and Regenerative Medicine, Department of Plastic and Hand Surgery, University Hospital of Erlangen, Friedrich-Alexander University Erlangen-Nürnberg (FAU), 91054 Erlangen, Germany

**Keywords:** bioprinting, skin graft, skin substitution

## Abstract

Human skin is particularly vulnerable to external damaging influences such as irradiation, extreme temperatures, chemical trauma, and certain systemic diseases, which reduce the skin’s capacity for regeneration and restoration and can possibly lead to large-scale skin defects. To restore skin continuity in severe cases, surgical interventions such as the transplantation of autologous tissue are needed. Nevertheless, the coverage of larger skin defects caused by severe third-grade burns or extensive irradiation therapy is limited due to the depletion of uninjured autologous tissue. In such cases, many of the patient’s epidermal cells can become available using biofabricated skin grafts, thereby restoring the skin’s vital functions. Given the limited availability of autologous skin grafts for restoring integrity in large-scale defects, using bioprinted constructs as skin graft substitutes could offer an encouraging therapeutic alternative to conventional therapies for large-scale wounds, such as the transplantation of autologous tissue. Using layer-by-layer aggregation or volumetric bioprinting, inkjet bioprinting, laser-assisted bioprinting, or extrusion-based bioprinting, skin cells are deposited in a desired pattern. The resulting constructs may be used as skin graft substitutes to accelerate wound healing and reconstitute the physiological functions of the skin. In this review, we aimed to elucidate the current state of bioprinting within the context of skin tissue engineering and introduce and discuss different bioprinting techniques, possible approaches and materials, commonly used cell types, and strategies for graft vascularization for the production of bioprinted constructs for use as skin graft substitutes.

## 1. Introduction

**Figure 1:**
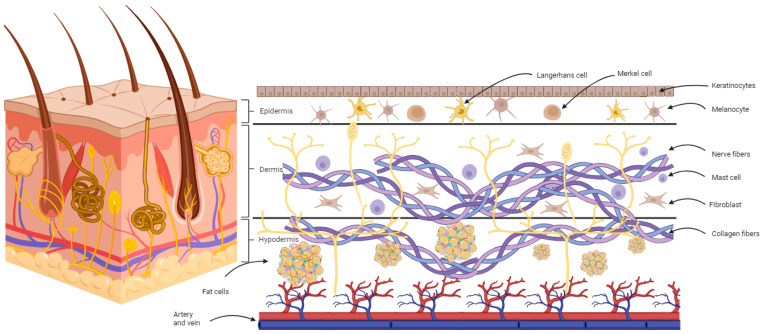
Schematic representation of the composition of human skin, including the epidermis, which contains keratinocytes, melanocytes, Langerhans cells, and Merkel cells; the dermis, which contains collagen fibers, fibroblasts, and mast cells; and the hypodermis, which contains the skin’s blood supply, fat cells, and nervous plexus (created using BioRender.com, accessed on 14 September 2023).

The skin is the largest organ of the human body. It represents the body’s outermost connection with the natural environment as well as with diverse pathogens. Among its other functions, it serves as a protective layer, with its integrity being essential for survival. It is composed of an outer layer, the epidermis, which is supplied by an inner layer, the dermis. Both layers are supplied by the hypodermis, which contains a rich vascular supply network and mesenchymal stem cells that are essential for the skin’s nutritional support and regeneration. The epidermis provides a tough outer mantel represented by the complex interdigitation of cytoplasmatic membrane folds in the spinous layer underneath the granular and cornified layers [1,2,3] (Figure 1).

Through acute and chronic damage to the skin, elementary reparatory mechanisms consisting of overlapping phases of hemostasis, inflammation, proliferation, and cell maturation are initiated. The blood flow toward the damaged area is increased, allowing the invasion of macrophages, mesenchymal stem cells, fibroblasts, and endothelial cells and their subsequent activation [4]. Because of the perpetual balance between proliferation and renewal, human skin is particularly vulnerable to damaging influences such as irradiation, extreme heat or cold, chemical trauma, or certain systemic diseases such as diabetes, which reduce the skin’s capacity for regeneration and restoration. 

A possible consequence of the aforementioned damage may be extensive breaks in the skin’s continuity [5,6]. When human basal keratinocytes are damaged, there is a subsequent disruption of the balance between cell death and cell production. Consequently, free radicals are released, which can cause damage to the nuclear and mitochondrial keratinocyte DNA and, subsequently, produce inflammatory changes within the skin. This may lead to a permanent disruption of the skin’s wound-healing capacity [7,8,9]. The treatment of these cutaneous manifestations is adjusted according to their severity. Erythematous lesions or localized breaks in skin continuity can be treated with topical dressings or steroidal ointments. In more severe cases, patients suffer from skin necrosis or ulceration. To restore skin continuity in such cases, surgical interventions such as the transplantation of autologous tissues, namely, skin grafts and pedicles or free flaps, are needed. Currently, even though skin grafts and pedicles or free flaps show high success rates, they continue to rely on an adequate blood supply and tissue oxygenation. Additionally, the challenges we face during the treatment of chronic and extensive wounds include patient-related factors such as diabetes, infections, systemic diseases, and damage from radiation or infection. There is also a risk of impaired or prolonged wound healing at the donor site, which can result in long-term donor-site morbidity or inadequate functional recovery over time [10,11]. In addition, after large-scale third-grade burns or extensive irradiation therapy, defect coverage can be limited due to the depletion of uninjured autologous tissue [12].

**Figure 2:**
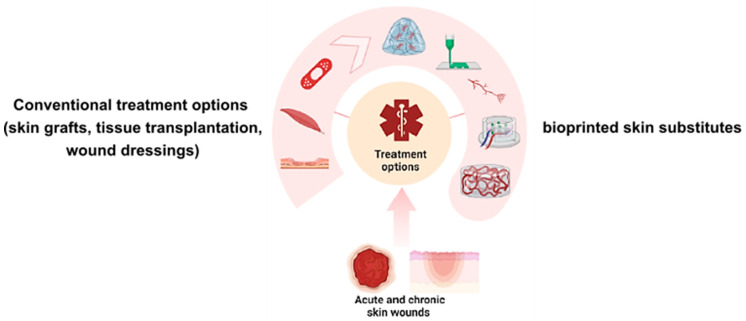
Schematic representation of current conventional treatment options for large-scale skin defects as well as a schematic representation showing bioprinted skin substitutes.

Optimally, large amounts of the patient’s epidermal cells would be available without distinct donor site morbidity to restore the skin’s vital function in cases that do not allow for an autologous transplantation [13]. One possible strategic solution in the preceding cases would be the development of bioprinted constructs containing the patient’s own cells as skin substitutes. These bioprinted constructs would ideally allow water and vapor transmission and provide sufficient stability for proper integration of the construct into the patient’s skin (schematic presentation Figure 2) [14]. 

Advances in tissue engineering in the past decades have already led to many commercially available mono- or multilayered, biological, and synthetic new skin substitutes. For example, cultured epithelial sheet grafts [15,16,17] by themselves, on carriers, or on suspensions of cultured keratinocytes [13,18] have been integrated into epithelial autografts and successfully used in burn treatment since the early 1980s [18]. Before the advent of bioprinting, various elaborate research models attempted to imitate the natural process of skin reconstitution by combining cell culture techniques with allogenic dermal grafts to mimic 3D skin substitution. This concept was experimentally derived from clinical techniques using cultured human keratinocytes in a fibrin sealant (KFGS) together with glycerol-preserved allogenic rat skin in a full-thickness wound in a nude mouse. In this way, a 3D skin equivalent was created experimentally with the induction of neodermis under fully re-epithelialized wound surfaces. Acellular synthetic skin substitutes mostly function as a barrier to avoid extensive fluid loss [19]. 

The use of tissue-engineered skin substitutes, for example, cultured epithelial sheet grafts, is still limited by insufficient dermis elasticity and regeneration of skin appendages as well as long-term postoperative scarring [20]. Ideally, an exemplary skin graft substitute would allow for sufficient nutrient diffusion into the graft, show healing and revascularization rates equal to or better than those of full-thickness skin grafts, and would have minimal donor site morbidity. Bioprinted constructs for skin graft substitution can more easily mirror an ideal framework for optimal cell ingrowth due to their 3-dimensional (3D) structure, which can be adapted to the shape and depth of the wound surface. Hydrogels, made of different natural or synthetic polymers, can be blended with cells or organoids and certain active substances that are best compatible with the relevant cell type. Firstly, they can mimic the extracellular matrix and secondly, allow the diffusion of nutrients, cytokines, and growth factors through the construct. This diffusion is compromised and insufficient in commercially available skin substitutes [21]. In addition, cells in a 3D matrix can form cell junctions and clusters in different layers leading to the production of tissue-like structures that interact with their environment. In combination with the proper structural and 3D orientation of the cells and biomaterials, this mimics complex in vivo conditions and facilitates the possible integration of a biofabricated skin graft into the human organism. Moreover, cell survival and differentiation, as well as gene and protein expression and the reaction to certain active substances of cells in hydrogels, are enhanced [21,22,23]. 

Bioprinted constructs for skin graft substitution can not only aid in the treatment of large-scale wounds but also provide an excellent environment for the facilitation of human wound healing. In summary, bioprinted constructs as skin graft substitutes may well serve as a precisely personalized replacement, which might be capable of reducing rejection risks and decreasing the patient’s waiting time, as well as the regeneration period in the near future. To provide a better understanding of the bioprinting process, we aim to review different aspects related to bioprinted constructs as a substitute for skin grafts and to introduce currently used techniques in the bioprinting process for skin substitutes, biomaterials, and their mechanical properties. In addition, we also review different cell types used for biofabricated skin equivalents along with future possibilities of further development and current challenges. 

## 2. Bioprinting Technique

Bioprinting itself can be described as a three-dimensional (3D) manufacturing process, incorporating living cells and/or their aggregates into organic or inorganic models [24]. Stereolythography for the 3D-printing process of objects was invented in the early 1980s. In 1988, Klebe printed 3D fibronectin-patterned substrates for cell adhesion on which cells were seeded to obtain cell sheets of the desired geometry. Laser-guided direct writing, whereby cells in suspensions are guided onto a substrate, was developed by Odde et al. in 1999. The inkjet bioprinter was developed by Wilson and Boland in 2004 [25]. Since its early beginnings in 1988, the bioprinting process has evolved to provide a new approach to the fabrication of biological constructs in a desired 3D pattern. This is achieved using an exact layer-by-layer aggregation or the volumetric bioprinting of biomaterials in the intended pattern [26]. In recent years, new techniques for enabling a precise printing process with minimal damage to the printed cell type have emerged. Most printing approaches contain a computer-designed template as a guide for the bioprinting hardware, which most often enables printing resolutions of 10–1000 µm with precise control of cell distribution. The most successful techniques comprise extrusion-bioprinting, laser-assisted-bioprinting, and inkjet-bioprinting as well as stereolithography [27,28].

Inkjet bioprinting achieves resolutions of 20–100 µm using a noncontact printing technology either with the application of a piezoelectric actuator modulated with an electric pulse or using thermoelectrical-modulated inkjet bioprinting. The latter is more suitable for printing processes involving living cells [28]. With both techniques, a continuous flow or an on-demand outflow can be achieved. Advantages of this technique include cost efficiency, high resolution, and higher cell viability compared with other technologies. However, a major disadvantage is the requirement of a liquid bioink with a low viscosity for the appropriate ejection [28].

Laser-assisted bioprinting is based on laser-induced transfer technologies. A donor layer, containing an upper energy absorbing layer and a lower layer of bioink, reacts to laser stimulation. A portion of the donor layer is evaporated during the printing process, as small areas of the absorbing layer are stimulated. This leads to the propulsion of the bioink and the collection of the substrate on a receiving substrate. Thereby, there is no direct contiguity between the hydrogels used as a bioink and the printing dispenser, causing a reduction in mechanical stress to the printed cells. Consequently, cell viability lies around >95%. The usage of this technique allows for the printing of bioinks with a higher viscosity. However, laser-assisted bioprinting is associated with high costs. Any negative influences of the technique on the cells are yet unknown [26,27,29].

Extrusion-based bioprinting is conducted with a pneumatic or mechanical force by which the ink is pushed through the nozzle onto the receiving building platform. During the extrusion in a state of low viscosity, the bioink remains stable until deposition with an additional crosslinking [29]. This technique is the foundation of most commercially available bioprinters. Materials with high cell density can be printed in a wide range of biomaterials. It also enables the production of continuous lines in multiple layers and forms. Compared with other techniques, the printing speed of extrusion-based bioprinting is rather slow. However, because of the compression mechanism used in the printing process, there is a higher chance of cell damage within the hydrogel, leading to cell viability of 40–80% [27,28,29].

Another method for creating bioengineered skin substitutes is volumetric bioprinting. With this technique, living tissue constructs can be printed within a short duration. This approach allows for the creation of entire cell-laden constructs at once using optical tomography. Positive and negative features are sculpted into hydrogels using a laser beam at different wavelengths directed at hydrogels inside printing vials. Thereby, an exact degree of biomimicry can be achieved. Nevertheless, this process is limited through cell-mediated light scattering, which negatively affects printing resolution. Volumetric bioprinting has yet to be tested as a possible method for the biofabrication of skin substitutes [30].

## 3. Biomaterials

**Figure 3:**
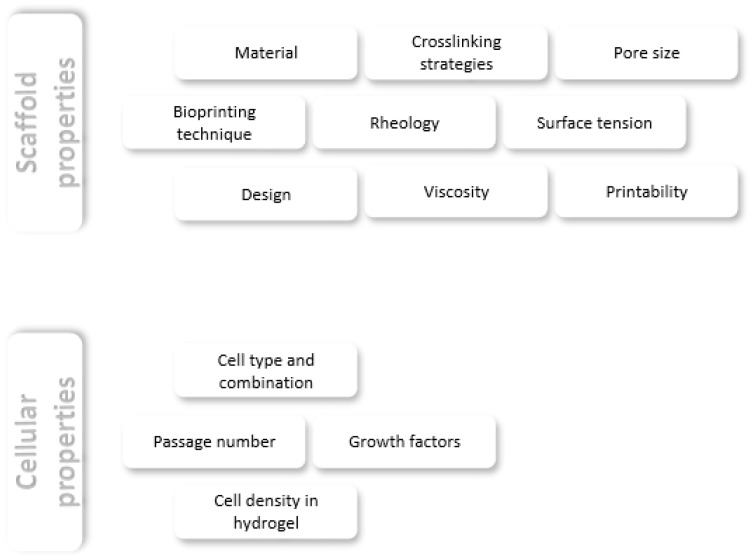
Schematic representation showing the development process for biofabricated skin substitutes containing necessary cellular and construct properties.

In order for the previously described techniques to result in a stable and accurate biofabricated skin construct, certain parameters have to be met to provide structural and rheological properties that are sufficient for the bioprinting process and allow good cellular interactions (Figure 3).

**Figure 4:**
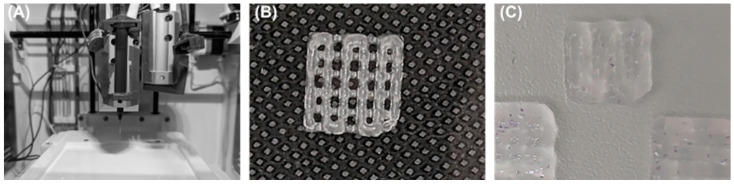
(**A**) Bioprinter with cartridge. (**B**) Bioprinted construct after successful printing process with an alginate-gelatin hydrogel. (**C**) Three specimens printed with laminin/collagen IV-rich hydrogel with insufficient mechanical gel properties.

A fundamental prerequisite is the printability of the hydrogel. Since the bioink is in direct contact with cells, it needs to provide the necessary physical support for the cells to interact, grow, and proliferate and simultaneously have the optimal viscosity or rheology, which is ideally shear thinning, and suitable crosslinking properties. The hydrogel surface tension needs to be sufficient for the preservation of the vertical direction and shape-fidelity of the construct, as demonstrated in Figure 4B). 

With insufficient surface tension, a construct’s initial design grows indistinct shortly after the printing process (Figure 4C). Furthermore, the substrate’s coherence influences cell viability by enabling cell adhesion and anchorage through the dynamic interface between the cells and the hydrogel [31]. The choice of biomaterial is crucial for cell survival. Ideally, the cells are stimulated by the biomaterial or by the growth factors incorporated inside of it. The material needs to be compatible with the dermal and epidermal cells, encouraging an appropriate host response after cell seeding and avoiding immunogenic reactions. It needs to have a suitable degradation rate with no toxic byproducts released during the process. Under optimal conditions, an environment is created that protects the cells during the process of bioprinting and promotes their natural function and proliferation during the cultivation process [27,32].

Human skin cells should be cultivated in a bioink containing naturally occurring biomaterials that display natural components of the human epidermis and dermis. This bioink should ideally include hyaluronic acid, collagen, albumin, or laminin. 

Collagen resembles the main structural protein of the extracellular matrix and possesses tissue-matching physiochemical properties, provides excellent biocompatibility, and promotes cell adhesion, migration, and proliferation. Gelatin, which is the denatured form of collagen and can be derived from bones, animal skin, or tendons using acidic or basic hydrolysis processes, shows thermoresponsive properties and can be physically cross-linked using thermal gelation. This temperature-induced gelation is reversible. To avoid this, gelatin has been altered with photopolymerizable methacrylol groups, namely, GelMA, to permit cross-linking, e.g., with a photoinitiator and UV light [33]. Thereby, construct stability and mechanical strength are superior to hydrogels based solely on collagen and gelatin. Stratesteffen et al. demonstrated enhanced cell spreading, excellent cell viability, and the formation of distinct capillary structures inside hydrogels combining collagen and GelMA [34].

Hyaluronic acid is a non-sulfated glycosaminoglycan that is found in the extracellular matrix and shows viscous properties. These viscous properties increase and decrease proportionally to the hyaluronic acid molecular weight [35]. Like gelatin, hyaluronic acid can be methacrylated to produce a photo-cross-linkable form [33]. 

Nonetheless, they show deficient mechanical properties and decreased reproducibility combined with high material costs [36,37]. Synthetic biomaterials, such as polyglycolic acid or synthetic polymer synthesized using ethylene oxide polymerization (PEG), can be produced with different chain lengths including linear or multi-armed structures. They are mastered better regarding their chemical and mechanical features. PEG at high molecular weights is non-cytotoxic and non-immunogenic but has been shown to decrease rates of cell attachment. There may also be some possible negative influence of synthetic biomaterials such as PEG on the cells included in the construct, which is yet not properly understood [38,39].

No bioink by itself has ideal preconditions as a single biomaterial. To avoid drawbacks caused by negative side effects resulting from the use of single-material-based bioinks, multicomponent hydrogels may be utilized. Thereby, favorable properties of natural, cell-friendly biomaterials such as increased cell migration, proliferation, and adhesion of collagen-derived gelatin, hyaluronic acid, or fibrin, necessary for good cellular interactions, are combined with positive mechanical properties of synthetic bioinks. By creating a mixture of different hydrogels, the elasticity, printability, and thermal stability of a bioink can be altered according to the acquired proportions [40]. 

## 4. Cell Types

The choice of skin cells used in bioprinted constructs for skin graft substitution should model itself on the natural skin composition. Cells from the hypodermal, dermal, and epidermal components should be included equally to optimize the construct stability and to produce adequate biomimicry of human skin. The skin cells mainly involved in the wound-healing cascade are fibroblasts, keratinocytes, endothelial cells, and stem cells. 

Fibroblasts synthesize collagen and aid in the incorporation of collagen type III naturally occurring in human skin. They also secrete growth factors, glycosaminoglycans, proteoglycans, and cytokines, support the angiogenesis process, and are crucial for the deposition and organization of the extracellular matrix. They provide structural integrity and stability to the construct as well as after implantation in vivo to the damaged tissue. Keratinocytes activate macrophages and neutrophils and induce re-epithelialization. Stem cells, such as mesenchymal stem cells found in the hypodermis, have the ability to differentiate into adult cells, influence inflammatory processes, and promote angiogenesis [41]. All these different cell types should be combined due to their synergistic interaction, which creates an excellent prerequisite in the process of wound healing. While designing the 3D shape and processing, the natural structural orientation of the cells needs to be taken into consideration. The superficial layers of the construct should contain keratinocytes and melanocytes to best emulate the epidermis and initiate the healing process and re-epithelialization. Fibroblasts and mast cells should be positioned in deeper layers of the construct, such as a collagen-containing layer, to mimic the dermal component. Yet, a continuous crosswalk between keratinocytes and fibroblasts should occur throughout the hydrogel to allow cytokine and growth factor signaling [42]. 

Keratinocyte proliferation is promoted by the secretion of keratinocyte growth factor from fibroblasts. Additionally, fibroblast proliferation is supported by the secretion of proangiogenic molecules from keratinocytes [43]. Adipose-derived stem cells (ADSCs), preadipocytes, endothelial cells, and perycites should be deposited into deeper layers to mimic the hypodermis [44]. ADSCs aid in the repair of damaged cells by proliferating and differentiating into adult cell types. Endothelial cells and pericytes promote the neovascularization of the construct. They regulate skin homeostasis in the wound-healing process and increase the production of granulation tissue and the formation of the extracellular matrix [45].

## 5. Vascularization Strategies

Even though there are many approaches to mimic the specific structure and cellular functionality of the multi-layers of the human skin, for a fully functional bioprinted construct, the skin’s blood supply originating in the hypodermis needs to be mimicked as well. Adequate vascularization avoids hypoxic and ischemic tissue as well as wound dehiscence and ulcer reoccurrence. Simultaneously, it promotes cell proliferation and proper graft incorporation, thus promoting long-term survival. On the contrary, long-term tissue hypoxia leads to tissue loss and a higher risk of wound infection [46,47].

**Figure 5:**
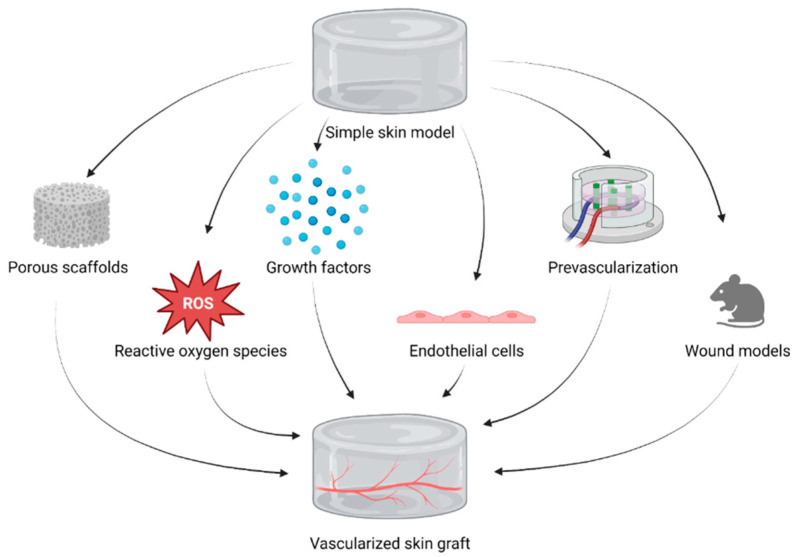
Schematic representation of vascularization strategies for bioengineered skin grafts. (Created with BioRender.com, accessed on 14 September 2023).

To achieve vascularization in tissue-engineered skin substitutes, there are several possible techniques available (Figure 5). 

Firstly, an approach enhancing angiogenesis may be used. Hereby, the formation and growth of newly formed blood vessels is promoted. A pore size smaller than 200 µm was proven to encourage the formation of a network of small-sized vessels with high density. The pores need to be interconnected and always greater than 40 nm [48]. Angiogenesis can secondly be stimulated indirectly by reactive oxygen species, which, in small amounts, can trigger cell signaling pathways that enhance the secretion of angiogenic factors, such as platelet-derived and vascular endothelial growth factors. Reactive oxygen species can therefore be stimulated by the graft stimulation with ionized low-pressure gas to break the covalent bonds on the graft’s outer face or by low levels of laser irradiation [49,50,51]. Another possible strategy is pre-vascularization, a process including the modification of the graft with growth factors and consecutively seeding it together with human endothelial colony-forming cells. This method enables a better, quicker, and more appropriate vascularization and graft incorporation. The aim is to develop vessel-like structures inside the tissue-engineered skin substitute before transplantation [48]. 

**Figure 6:**
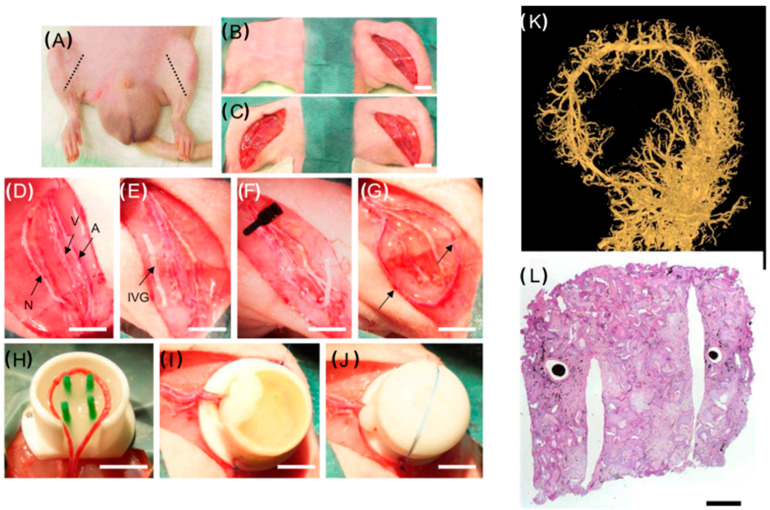
Arterio-venous (AV) loop model with a visualization of graft vascularization: (**A**–**J**) Schematic representation of vascularization and implantation strategies for bioengineered skin grafts. A = femoral artery, V = femoral vein, N = femoral nerve, IVG = interpositional venous graft. Scale bar: 5 mm (**D**–**J**). (**K**,**L**) Micro CT and hematoxylin–eosin staining of construct implanted into an AV loop model for 6 weeks (scale bar = 1 mm). Adapted from Weigand, A., Beier, J. P., Arkudas, A., Al-Abboodi, M., Polykandriotis, E., Horch, R. E., Boos, A. M. The Arteriovenous (AV) Loop in a Small Animal Model to Study Angiogenesis and Vascularized Tissue Engineering. *J. Vis. Exp.* (117), e54676, doi:10.3791/54676 (2016) [54].

The formation of microvessels inside tissue-engineered skin substitutes can also be achieved with the implantation of the biofabricated skin graft into fully vascularized tissue. Possible implantation sites can constitute muscle pouches or subcutaneous compartments (Figure 6A–J) [52]. Laschke et al. induced pre-vascularization with construct implantation into the flank of donor mice for 20 days. Within this time arteriolar vessels and capillary and postcapillary venules had formed. After excision, the constructs containing the newly formed microvascular network were transplanted onto the dorsal skinfolds of isogenic recipient mice. Thereby, a significantly improved blood perfusion of the prevascularized construct was demonstrated, which led to an increase in the vascularization rates by 20 times compared with the non-prevascularized control group [52]. Alternatively, tissue-engineered skin substitutes can be implanted temporarily into the arterio-venous loop model. Thereby, the emergence of newly formed vessels as well as the proliferation of fibroblasts is stimulated proportionally to the implantation time [53].

Even though the ingrowth of vessels into the graft is possible, as demonstrated by Weigand et al. (Figure 6K,L) [54], their proper structural arrangement and the cultivation time needed for proper vessel ingrowth is a challenge yet to be solved. Ideally, the vascular network inside the construct would originate in the hypodermis and would be most pronounced there. A possible approach to solve this problem could be micropatterning a perfusable vascular network into the construct by printing out molds and microchannels into the deeper components of the construct, mimicking the dermis and hypodermis [55]. Mori et al. seeded endothelial cells into molded microchannels in a second construct fabrication step after proper keratinocyte and fibroblast differentiation. This resulted in a perfusable, physiological skin equivalent [55].

## 6. Challenges and Limitations in the Clinical Application of Bioprinted Constructs as Skin Graft Substitutes

Despite the medical technological progress over the past decades, there are still limits to the clinical application of biofabricated skin grafts. To achieve further advancements and reach a standardized clinical application, the complexity of human tissue needs to be approached, and the optimization of mechanical, technical, and cellular handling is needed. 

The main challenge is the proper cellular nutritional and oxygen supply of biofabricated skin substitutes. These are limited by using cell-seeded hydrogels, as they fail to mimic the structural and biological functions of the extracellular matrix to the necessary degree, possibly leading to graft failure in clinical applications. Also, the correct structural alignment of all structural components including the graft vessels presents a challenge that still needs to be solved. Despite possible and promising strategies to reach the proper graft vascularization mentioned above, researchers have not yet managed to mimic blood vessels of native skin, which results in vascularization rates lower than those of full-thickness skin grafts. In addition, the period needed to achieve proper graft vascularization in vitro or in vivo is still too great. Therefore, they are still subordinate to skin grafts and have not yet gained major clinical importance [56,57]. 

Maintaining the optimal conditions for cell survival during the printing process is an obstacle that still needs to be overcome. The optimal printing temperature and extrusion pressure needed for most hydrogels likely have a negative effect on cell survival. This can determine cell death and thereby lower the rates of successful implantation of the biofabricated skin graft [58]. There are also still setbacks related to material selection since the bioinks with the best biomimetic properties often lack the mechanical characteristics to provide sufficient strength to be printed solitarily. Bioinks such as hyaluronic acid, collagen, gelatin, and alginate that are best suitable for the cultivation of skin cells show low mechanical properties, such as shape fidelity [59,60]. 

To create a fully functional bioengineered skin graft, large quantities of cells are required. The amount and quality of these cells make them time-consuming to harvest and cultivate. In comparison with conventional transplantation of autologous tissue, this method prolongs the patient’s treatment period. A large number of cells might be difficult or impossible to obtain in certain cases, such as burn injuries, where a large total body surface area is affected. To overcome these issues the production and storage of large numbers of biofabricated skin substitutes would be needed. However, long-term storage is impeded by the limited lifespan of cells [61]. The time-consuming cultivation period, the maintenance, the highly specialized staff required for the production, and the assimilated transportation of bioprinted skin substitutes all immensely increase the manufacturing costs [57]. Regardless of past successes, the reproducibility of bioprinted skin substitutes is still limited, and the 3D bioprinting process is not fully optimized yet. This causes safety and legal problems in the clinical application of biofabricated skin substitutes, limiting their standardized application and reimbursement from patient insurance providers [62].

## 7. Comparison to Other Methods for Bioengineered Skin Graft Fabrication and Current Clinical and Experimental Applications 

**Figure 7:**
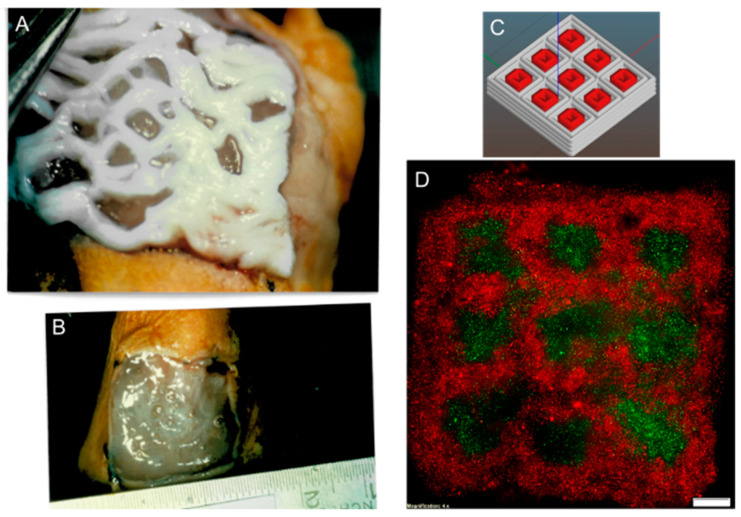
Human cultured keratinocytes on various carrier systems. (**A**,**B**) Keratinocyte cultured in fibrin sealant on an artificial wound in a BALB/c nude mouse model (d0) covered with glycerol-preserved rat skin matrix. (**C**) Schematic representation showing the design of a bioprinted construct containing keratinocytes (grey) and ADSC (red). (**D**) Bioprinted construct containing keratinocytes (red) and ADSC (green) stained with DiI = red (keratinocytes) and Oregon green = green (ADSC), scale bar = 1 mm.

Bioprinted constructs as skin graft substitutes in theory may have the possibility to overcome well-known and unsolved limitations of autografts and allografts for the coverage of skin defects. The bioprinting of multidimensional whole skin substitutes could help to accelerate wound healing as they are capable of emulating the stratified epidermis [63]. Nevertheless, it should be questioned critically whether currently existing biofabricated alternatives such as the use of nanoparticles, nanofibers, cell-laden commercial skin templates, or cell-laden hydrogels still are superior to the bioprinting process (representative images in Figure 7A,B). 

With the use of cell-laden skin templates, such as cultured epithelial sheets or dermal or epidermal templates, autologous keratinocytes can be cultivated on pre-existing membranes such as porcine dermis or hyaluronic acid membranes [64,65]. This provides the advantage that the patient’s own cells can be applied. However, with the use of prefabricated membranes, there is a substantial time reduction in the treatment period. Additionally, the choice of material can be adapted to best fit the depth of the patient’s wound. For example, in defects reaching the dermal skin component, membranous dermal substitutes can be chosen to replace the dermal skin component and can be combined with human autologous keratinocytes, resembling the epidermal component. Boyce et al. demonstrated the formation of hemidesmosomes, extracellular matrix, and banded collagen on the surface of a graft between epidermal cells and the artificial dermal membrane [66]. Prenosil and Kinooka described an automated membrane bioreactor for producing autologous wound dressings. This automated membrane reactor shortened the time of transplantation to a two-week interval at a significantly lower cost. Their system comprises a computer-controlled bioreactor in which medium changes during keratinocyte culture are fully automated. The use of bioreactors to automate the fabrication processes of biofabricated constructs as skin graft substitutes has the potential to decrease costs, increase reproducibility, and permit more efficient regulation of biofabricated skin substitutes anatomy and physiology. At the same time, Prenosil et al. also hypothesize that the risk of human error can be largely eliminated [67]. 

Another possible therapeutic strategy is the combination of bioengineered skin substitutes with meshed skin grafts. It has been shown that tissue-engineered skin substitutes, composed of cultured autologous fibroblasts and keratinocytes inoculated on a collagen-glycosaminoglycan sponge, can effectively aid in the treatment of large-scale wounds in combination with meshed autologous split skin grafts [68].

Furthermore, keratinocytes can be applied to large-scale wounds in a sprayed cell suspension. For this method, fibrin was demonstrated to be a suitable biological carrier for cultured epithelial cells both experimentally and clinically. Cells can be applied using a pneumatic atomizer or other spraying devices. Thereby, an equal application of the suspension is provided, and larger wound surfaces can be covered easily. This technique has been proven to be more efficient regarding a homogenous and time-efficient utilization when compared with the application of the cellular suspension as a droplet [13,69]. With this technique, there is a possibility of simultaneous implantation of multiple cell types; however, there is no possibility of achieving a proper structural composition, for example, dividing dermal and epidermal skin components.

A successful biofabrication of skin graft substitutes was achieved by Moiemen et al. They reported the treatment of a burn victim with 95% of body surface area affected with a bioengineered autologous dermo-epidermal skin analog. Dermal fibroblasts were seeded on a collagenous hydrogel, and after proper cell attachment, keratinocytes were seeded on top of the collagenous hydrogel to represent the dermis. In their case report, they delineate the obtainment of a 1904 cm^2^ bioengineered skin analog after the harvest of a skin biopsy with a surface area of 8.4 cm^2^ [61]. 

Despite the existing limitations in the clinical application of bioprinted constructs as skin graft substitutes, there were several reports of their successful clinical use. Jorgensen et al. [70] demonstrated wound closure with re-epithelialization after the utilization of a fibrinogen and hyaluronic acid-based bioprinted skin equivalent containing human keratinocytes, melanocytes, fibroblasts, dermal papilla cells, and endothelial cells. The bioprinted skin group evaluated in their research showed significantly superior wound healing when compared with a hydrogel without cells as well as to an untreated control group. These findings are in concordance with Kim et al., who evaluated a 3D prevascularized skin model in an in vivo setting and demonstrated accelerated wound healing with re-epithelialization and neovascularization of the construct [71]. Albanna et al. demonstrated superior wound healing in a mouse and porcine model after treatment with inkjet-based 3D bioprinted constructs containing fibrinogen, thrombin, and collagen. During the histological analysis of the wounds after treatment with 3D bioprinted constructs, they demonstrated a quicker epithelialization, earlier formation of a papillary layer, and a dermal layer with large blood vessels incorporated inside [72]. Ideally, the epidermal, dermal, and hypodermal skin components are mimicked by the bioink and the cells it contains, as all components are needed to create a viable skin equivalent [73]. Skin cells such as fibroblasts and keratinocytes are optimally printed in a bioink containing collagen and gelatin, yet they should be separated into dermal and epidermal layers. The inclusion of fibroblasts is crucial for the correct resemblance of the dermal component of the bioprinted construct as a skin graft substitute. Hyaluronic acid was proven to increase the proliferation of fibroblasts and keratinocytes. On the one hand, these natural biomaterials have a positive interaction with cells and, on the other hand, possess anti-inflammatory properties to convey skin regeneration and wound healing [41,74]. The addition of the dermal component to bioprinted skin grafts has proven to increase this stability and also lead to better surgical graft handling [75]. 

## 8. Conclusions

Given the limited availability of autologous skin grafts for restoring integrity in massive burn wounds, bioengineered skin substitutes offer an encouraging therapeutic alternative to the conventional therapy of large-scale wounds such as transplantation of autologous tissue. By means of layer-by-layer aggregation or volumetric bioprinting, inkjet bioprinting, laser-assisted bioprinting, or extrusion-based bioprinting, skin cells are deposited in a desired pattern (Figure 7C,D). With the high level of flexibility in the process of construct fabrication, complexities such as the width and depth of wounds can be balanced, and complications in the healing process can be avoided. Yet, there still exist multiple problems that need to be solved before the standardized clinical use of bioprinted skin substitutes is possible. The graft’s structural and compositional structure needs to be better adapted to human skin. Vascularization strategies need to be improved so that graft vascularization is at least as fast as the in vivo vascularization of full-thickness split skin grafts. Furthermore, production costs and time need to be lowered to allow for clinical use. After solving these problems, over the long term, the treatment of chronic wounds and burns can be optimized and patient survival and long-term health status can be improved, at best. Bioprinted constructs as skin substitutes can solve problems such as long-term donor site complications and lack of autologous material needed for transplantation as well as with the proper 3D design improve functional outcomes. In addition, they lead to a decrease in graft rejection rates by the provision of 3D skin-like structures, which are optimally adjusted to each patient since they are based on the patient’s own cells. Furthermore, it allows the mutual implantation of multiple cell types. The usage of a bioink, which is harmonized to provide optimal conditions for the best cell survival and proliferation, might favor the healing process and could lead to an improvement in the current standards for the treatment of large-scale or chronic wounds in the future.
